# 578 A Cross-Sectional Study of Sex, Race, and Ethnic Representation in Burn Clinical Trials

**DOI:** 10.1093/jbcr/irae036.212

**Published:** 2024-04-17

**Authors:** Sara Sheikh-Oleslami, Brendan Tao, Bettina Papp, Shreya Luthra, Anthony Papp

**Affiliations:** The University of British Columbia, North Vancouver, BC; University of British Columbia, Vancouver, BC; Douglas College, Burnaby, BC; Vancouver General Hospital, Vancouver, BC; The University of British Columbia, North Vancouver, BC; University of British Columbia, Vancouver, BC; Douglas College, Burnaby, BC; Vancouver General Hospital, Vancouver, BC; The University of British Columbia, North Vancouver, BC; University of British Columbia, Vancouver, BC; Douglas College, Burnaby, BC; Vancouver General Hospital, Vancouver, BC; The University of British Columbia, North Vancouver, BC; University of British Columbia, Vancouver, BC; Douglas College, Burnaby, BC; Vancouver General Hospital, Vancouver, BC; The University of British Columbia, North Vancouver, BC; University of British Columbia, Vancouver, BC; Douglas College, Burnaby, BC; Vancouver General Hospital, Vancouver, BC

## Abstract

**Introduction:**

The demographic proportions of plastic surgery trials approximating real-world disease are not well studied. Judicious trial representation is essential in treatment evaluation across diverse patient populations. Herein, we investigate sex, racial and ethnic disparities in patient enrollment across burn trials.

**Methods:**

Cross-sectional analysis of participants enrolled in high-quality, reduced risk of bias, randomized controlled trials (RCT) registered on clinicaltrials.gov under the query “burn”. Completed RCTs reporting minimum two demographic groups, employing double masking or greater, with results accessible through registry or publications were included. Trial characteristics (country, site location, year, study phase, masking) and demographic data (sex, race, ethnicity) were collected. The Global Burden of Disease database provided sex-based burn disease burdens.

**Results:**

The primary outcome was the population-to-prevalence ratio of enrolled female participants. Secondary outcomes included representation of racial and ethnic populations as related to blinding, phase, and study/sponsor locations. Of 546 trials, 41 were included (2919 participants). All reported sex demographics, females comprising 37.02% of all participants (PPR=0.71, 95%CI [0.59,0.82], likely indicating underrepresentation against their empiric disease burden). Only 7 and 9 reported ethnicity and race, respectively, although not comprehensively. Caucasians and Black persons comprised 57.52% and 21.80% of participants, respectively, while only 9.80% had Hispanic/Latino ethnicity.

**Conclusions:**

Females are likely underrepresented in high-quality, US-registered burn trials, unreflective of their real-world disease burden. Further, severe underreporting of race and ethnicity was noted. It is imperative that future trials collect and report demographic data, namely race and ethnicity, and attempt enrolment of diverse demographics and equitable populations for promotion of study generalizability of efficacy data across relevant populations.

**Applicability of Research to Practice:**

As there is natural variation in the effect of various medications, treatments, or medical products used amongst different sexes and diverse races due to factors such as differing physiological and genetic characteristics, enrolment of participants reflective of the disease pool studied is essential in investigations of treatments or devices intended for clinical practice. Failure to encompass all populations, due to lack of diversity in undergoing treatments further compounds healthcare disparity.

